# The Effects of Online Mindfulness-Based Intervention on Posttraumatic Stress Disorder and Complex Posttraumatic Stress Disorder Symptoms: A Randomized Controlled Trial With 3-Month Follow-Up

**DOI:** 10.3389/fpsyt.2022.799259

**Published:** 2022-03-30

**Authors:** Austeja Dumarkaite, Inga Truskauskaite-Kuneviciene, Gerhard Andersson, Evaldas Kazlauskas

**Affiliations:** ^1^Center for Psychotraumatology, Institute of Psychology, Vilnius University, Vilnius, Lithuania; ^2^Department of Behavioural Sciences and Learning, Linköping University, Linköping, Sweden; ^3^Department of Clinical Neuroscience, Karolinska Institute, Stockholm, Sweden

**Keywords:** posttraumatic stress disorder, complex posttraumatic stress disorder, mindfulness, internet intervention, effects, RCT, follow-up

## Abstract

**Objectives:**

Mindfulness-based interventions have recently been shown to be a promising option for treating posttraumatic stress. The current study aimed to investigate the effects of an online mindfulness-based intervention on ICD-11 posttraumatic stress disorder (PTSD) and complex PTSD (CPTSD) symptoms at a 3-month follow-up.

**Methods:**

An RCT design with three measurement points (pre-intervention, post-intervention, and 3-month follow-up) was used to investigate the effects of an 8-week online mindfulness intervention. In total, 53 traumatized young adults (*M*_*age*_ = 23.21, *SD*_*age*_ = 2.81; 84.9% female) participated in the study: 17 in the intervention group and 36 in the waiting list control group.

**Results:**

Intervention group and waiting list control group comparison revealed that the intervention was effective for reducing CPTSD disturbances in self-organization symptoms (*d* = −0.84 [−1.44; −0.24]), specifically, negative self-concept (*d* = −0.66 [−1.25; −0.07]) and disturbances in relationships (*d* = −0.87 [−1.47; −0.27]), at 3-month follow-up. There were no between-group effects for PTSD symptoms from pre-test to follow-up.

**Conclusion:**

This is one of the first RCT studies to report follow-up effects of an online mindfulness-based intervention for ICD-11 PTSD or CPTSD symptoms. Our study yielded that the effects of mindfulness-based internet intervention on CPTSD symptoms tend to retain over time.

**Trial Registration:**

This study was registered with ClinicalTrials.gov (NCT number: NCT04333667; https://clinicaltrials.gov/ct2/show/NCT04333667). Registered April 3, 2020.

## The Effects of Online Mindfulness-Based Intervention on Posttraumatic Stress Disorder and Complex Posttraumatic Stress Disorder Symptoms: Randomized Controlled Trial With 3-Month Follow-Up

Internet-delivered interventions for treating symptoms of posttraumatic stress disorder (PTSD) have been found to be effective in randomized controlled trials (1–4). Internet-delivered support in the context of posttraumatic stress is a promising solution since various barriers accessing face-to-face PTSD treatments have been recognized (5). Nevertheless, most internet-based treatments for PTSD have been based on trauma-focused cognitive behavioral therapy (3). Trauma-focused treatments seem to have limitations, such as high dropout rates (6) and persistent symptoms even after successfully finalized treatment (7). Moreover, a new diagnosis of Complex posttraumatic stress disorder (CPTSD) has been included in the 11th edition of the International Classification of Diseases (ICD-11) (8). For the diagnosis of CPTSD, in addition to the three core PTSD symptoms of (1) re-experiencing, (2) avoidance, and (3) sense of threat, three additional symptoms of disturbances in self-organization, particularly, (1) the affect dysregulation, (2) negative self-concept, and (3) disturbances in relationships, are a prerequisite (5). There is still little knowledge of the effects of various treatment strategies for CPTSD (9).

Mindfulness-based interventions have recently been shown to be a promising option for treating posttraumatic stress (10–15), including evidence from efficacy studies of internet-delivered mindfulness-based interventions on PTSD and CPTSD (16, 17). Mindfulness is described as the awareness that emerges through purposefully paying attention to the present moment and non-judgment to the unfolding experience (18). It is suggested that mindfulness diminishes physiological arousal, increases attentional control, and fosters acceptance of unwanted experiences; all these processes have the potential to interrupt the maintenance of PTSD (19). The existing evidence highlights the short-term effects of both face-to-face and internet-delivered mindfulness-based treatments on posttraumatic stress (10, 12–14, 16, 17). However, little is known about whether these effects tend to last after the intervention is over. To the best of our knowledge, only a few studies have explored the long-term effects of mindfulness-based interventions in PTSD, and the results suggest that effects tend to remain from one to 5 months after intervention (15, 20–23). However, most existing evidence is based on single group studies or small study samples. In addition, research has yet only explored face-to-face mindfulness-based therapies for PTSD.

The current study aimed to investigate the 3-month follow-up effects of an online mindfulness-based intervention on ICD-11 PTSD and CPTSD symptoms. Previously, randomized controlled trial comparing intervention group with a waiting list control group was carried out to assess short-term effects at post-treatment of 8-week mindfulness-based internet intervention on PTSD and CPTSD in a sample of young adults exposed to traumatic experiences. We have reported short-term effects with significant reductions in disturbances in self-organization symptoms at post-treatment, as well as significant improvement in positive mental health (16). In the current study, we sought to report the 3-month follow-up results by investigating intervention effects on PTSD and specific for CPTSD disturbances in self-organization symptoms in young adults’ sample comparing the intervention group with a waiting list control group.

## Methods

### Design

**FIGURE 1:**
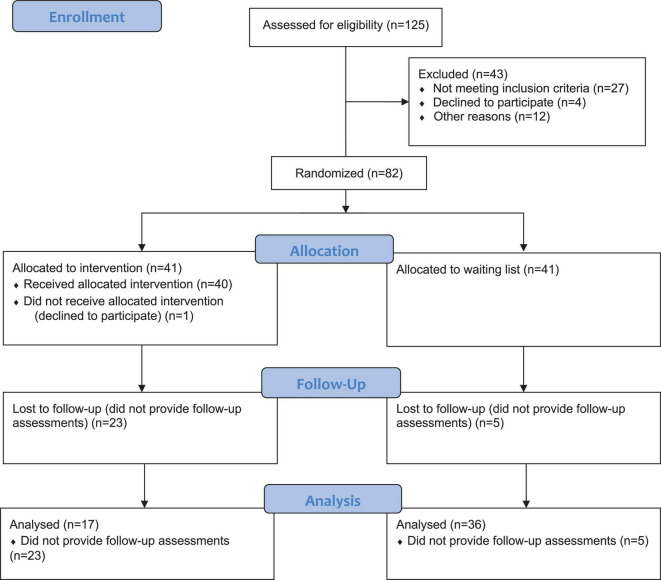
Flowchart of the Intervention.

A randomized controlled trial (RCT) design was used for the current study, comparing the intervention group with a waiting list control group. The data was collected at three measurement points: pre-intervention, post-intervention, and 3-month follow-up. The outcomes from pre-test to post-test have been reported previously (16). We investigated the effects of an online mindfulness-based intervention on PTSD and CPTSD-specific disturbances in self-organization symptoms at a 3-month follow-up. Participants were randomly assigned to the intervention group or the waiting list control group. Those allocated to the intervention group received the intervention immediately after randomization, whereas participants allocated to the waiting list control group received the intervention 5 months later. The intervention lasted for 8 weeks. T1, T2, and T3 were carried out at the same time in both groups. The flowchart of the study is presented in Figure 1. This study relied on self-reported measures using a secure web application (24). In the present study, all the data are reported following the CONSORT statement for reporting parallel group trials (25).

### Participants and Procedure

Participants and procedures have been described in detail previously (16). In brief, eligible participants were university students who were 18 years old or older; were fluent in Lithuanian; had access to a device with Internet; had experienced one or more traumatic events during their life; met the clinical significance criteria for PTSD, CPTSD, or disturbances in self-organization symptoms with or without functional impairment as measured with the International Trauma Questionnaire (26). The ongoing treatment was not an exclusion criterion. Participants who completed the pre-test measures and met all inclusion criteria were randomly assigned to either the intervention or waiting list control group.

Data were collected at three time points, March to April 2020 (T1), June to July 2020 (T2), September to October 2020 (T3). All data were collected online. The present study was approved by Psychology Research Ethics Committee (Reference No. 27-02-2020/36) and registered with ClinicalTrials.gov (NCT number: NCT04333667). Study participants gave active informed consent for participation before filling the pre-test questionnaires.

In the current study, 53 participants (*M*_*age*_ = 23.21, *SD*_*age*_ = 2.81; 84.9% female) who completed T1, T2, and T3 measures were included in the comparison analysis: 17 from the intervention group and 36 from the waiting list control group. In the intervention group, participants who logged in to the intervention site at least once were included in the analysis (29.5% logged in <10 times, 29.5% logged in 10–20 times, and 41.3% logged in >20 times; modules completed: *M* = 6.59, *SD* = 2.55). The power analysis revealed that the total sample of 46 participants was sufficient to detect the effect sizes of 0.35, indicating the differences between the two groups by using the multivariate three measurement points data analytic approach (given a significance level of 0.05 and a power of 80%). No differences were observed in demographic characteristics between the intervention group and waiting list control group at T1 in the current study sample, including age, partnership status, employment status, number of traumatic events, except for gender. There were more male participants in the intervention group as compared to the waiting list control group. Descriptive data on study participants at T1 are presented in Table 1. Additionally, the *t*-tests showed no significant differences between the intervention group and waiting list control group at T1 for PTSD (*t*(51) = 0.23, *p* = 0.822) and disturbances in self-organization (*t*(51) = −0.20, *p* = 0.841). Moreover, there were no differences between the two groups in terms of current visits to a psychologist (17.6% vs. 13.9%; χ2(1) = 0.13, *p* = 0.721) and current use of medicine due to mental health problems (23.5% vs. 33.3%; χ2(1) = 0.53, *p* = 0.468).

**TABLE 1:** Characteristics of the study participants (*n* = 53) at pre-test.

Variable	Intervention group (*n* = 17), *n* (%)	Control group (*n* = 36), *n* (%)	Significance statistics
**Gender**			
Male	6 (35.3%)	2 (5.6%)	χ^2^(1) = 7.97, *p* = 0.005
Female	11 (64.7%)	34 (94.4%)	
**Age**			
M (SD)	23.06 (2.84)	23.28 (2.83)	t(51) = 0.26, *p* = 0.794
Range	20–32	20–31	
**In partnership**			
Yes	6 (35.3%)	19 (52.8%)	χ^2^(1) = 1.42, *p* = 0.234
No	11 (64.7%)	17 (47.2%)	
**Employment**			
Yes	6 (35.5%)	17 (47.2%)	χ^2^(1) = 0.67, *p* = 0.413
No	11 (64.5%)	19 (52.8%)	
**Number of traumatic events**			
M (SD)	4.59 (2.03)	4.97 (1.96)	t(51) = 0.66, *p* = 0.514
Range	1–8	1–9	
**Visiting psychologist**			
Yes	3 (17.6%)	5 (13.9%)	χ^2^(1) = 0.13, *p* = 0.721
No	14 (82.4%)	31 (86.1%)	
**Using medicine due to mental health problems**			
Yes	4 (23.5%)	12 (33.3%)	χ^2^(1) = 0.53, *p* = 0.468
No	13 (76.5%)	24 (66.7%)	

The most prevalent traumatic event types were severe human suffering (exposure rate 75.5%), childhood physical abuse (exposure rate 50.9%), and physical assault (exposure rate 49.1%). A traumatic experience that affected the most, as indicated by the participants, was as follows: death of someone close (24.5%), physical abuse (17.0%), sexual trauma (15.1%), psychological abuse (15.1%), serious illness (7.5%), transportation accident (1.9%), several traumatic events (1.9%), and other traumatic events (17.0%).

There were no differences in demographic characteristics at T1 between retained and dropped out participants including gender (χ^2^(1) = 0.36, *p* = 0.546), age (*t*(80) = 0.35, *p* = 0.729), partnership status (χ^2^(1) = 0.98, *p* = 0.321), employment status (χ^2^(1) = 0.52, *p* = 0.470), and number of traumatic events (*t*(80) = −0.36, *p* = 0.717). Also, the *t*-tests showed no significant differences at T1 between retained and dropped out participants for PTSD (*t*(80) = −1.32, *p* = 0.190) and disturbances in self-organization (*t*(80) = 1.22, *p* = 0.226).

### Measures

#### Exposure to Traumatic Experiences

The DSM-5 Life Events Checklist (LEC-5) (27) was used to assess the lifetime exposure to 18 different traumatic experiences (e.g., natural disaster, sexual or physical violence, etc.) with one additional item assessing exposure to any other not-listed traumatic experience. Exposure level for each event was assessed by five types of responses: 1 (= “happened to me”), 2 (= “witnessed it”), 3 (= “learned about it”), 4 (= “not sure”), and 5 (= “does not apply”). In the present study, exposure to traumatic experience was considered if participants responded with either 1 (= “happened to me”) or 2 (= “witnessed it”). The Lithuanian version of LEC-5 has been validated and used earlier (28, 29).

#### Symptoms of Posttraumatic Stress Disorder and Complex Posttraumatic Stress Disorder

The International Trauma Questionnaire (ITQ) (26) was used to assess symptoms of PTSD and CPTSD. The ITQ is comprised of 18 items. The PTSD symptoms in the past month are assessed with the six symptom items on the three subscales (Re-experiencing, Avoidance, and Sense of Threat symptoms), with two items for each of the PTSD symptom clusters. The CPTSD-specific disturbances in self-organization symptoms in the past month are also assessed with the six symptom items on the three subscales (Negative Self-Concept, Affective Dysregulation, and Disturbances in Relationships symptoms) with two items for each of the disturbances in self-organization symptom clusters. Additional six functional impairment items assess how PTSD (three items) and disturbances in self-organization (three items) symptoms impaired functioning in the past month. Participants rated the ITQ items on a 5-point Likert scale ranging from 0 (= “not at all”) to 4 (= “extremely”). A score of ≥2 for at least one of the two items representing a particular PTSD and disturbances in self-organization symptom cluster indicates clinical significance based on the algorithm proposed by the ITQ authors (21). A probable PTSD diagnosis is given when all three PTSD symptoms are clinically significant and if they significantly impair functioning in at least one area of life. A probable diagnosis of CPTSD is given when all three PTSD symptoms are clinically significant, all three disturbances in self-organization symptoms are clinically significant, and if disturbances in self-organization symptoms significantly impair functioning in at least one area of life. In the study sample, Cronbach’s alpha of the scale and the subscales of PTSD and disturbances in self-organization symptoms separately at T1 were acceptable (α = 0.70, α = 0.73, α = 0.75, respectively).

### Intervention

The intervention has been described in detail previously (16). In brief, an online mindfulness-based intervention was developed for the present study. It was aimed at young adults with traumatic life events and PTSD or CPTSD symptoms. The intervention was designed as a self-help program (focusing on psychoeducation and mindfulness techniques training) with the possibility of messaging with a psychologist. The intervention consisted of eight modules: (1) Introduction, (2) Awareness and non-judgment of physical senses, (3) Physical senses in everyday life, (4) Awareness and non-judgment of thoughts, (5) Thoughts in everyday life, (6) Awareness and non-judgment of emotions, (7) Emotions in everyday life, and (8) Summary. The screenshot of the intervention is presented in Supplementary Figure 1.

### Data Analyses

To calculate intervention effects, we ran a series of multivariate repeated measures ANOVAs with time (T1, T2, and T3) as a within-subject factor and group (intervention group vs. waiting list control group) as a between-subject factor. First, we tested the intervention effects on PTSD and disturbances in self-organization symptoms using the sum scores for each measure. Then, we tested the PTSD symptoms subscales of Re-experiencing, Avoidance, and Sense of Threat. Also, we performed an analysis of the disturbances in self-organization symptoms subscales of Affective Dysregulation, Negative Self-Concept, and Disturbances in Relationships. We calculated within-group and between-group effect sizes. The between-group pre-test to follow-up effect sizes were calculated by using the mean difference from T1 to T3 in the intervention group and waiting list control group and the standard deviations of each group at T1 (30). The within-group pre-test to post-test, post-test to follow-up, and pre-test to follow-up effect sizes were calculated by using the means in each group at T1 and T2, T2 and T3, and T1 and T3, respectively, and standard deviations at each measurement point. Bias-corrected effect sizes (31) were reported. In all analyses, the magnitude of the effect expressed in *d* was interpreted according to Cohen (32), that is, 0.50 = medium effect, and 0.80 = large effect.

## Results

**FIGURE 2:**
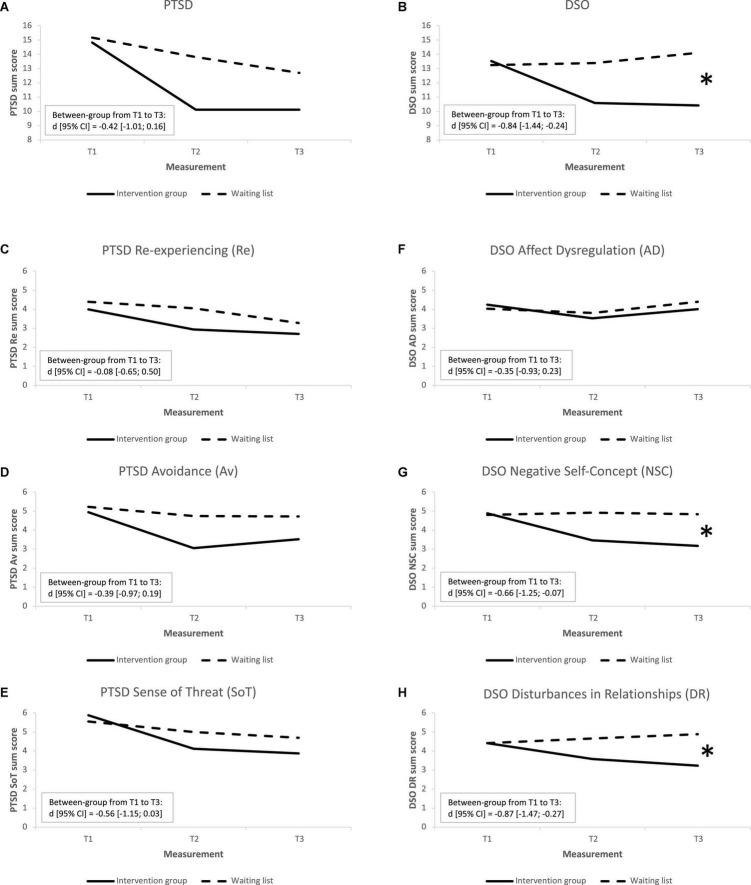
Trajectories of change of intervention outcomes in the IG (*n* = 17) and WL control group (*n* = 36). **(A)** PTSD – posttraumatic stress disorder, **(B)** DSO – disturbances in self-organization, **(C–E)** PTSD symptom clusters, **(F–H)** DSO symptom clusters. * Between-group effect size from T1 to T3 is significant.

At the multivariate level, ANOVA analyses revealed a significant difference in change of PTSD and disturbances in self-organization sum scores over time between the intervention group and waiting list control group (*F*(4, 202) = 3.11; *p* = 0.016; Wilks‘ λ = 0.80). At the univariate level, we found a significantly higher decrease in the disturbances in self-organization score in the intervention group compared to the waiting list control group (*F*(2) = 4.90; *p* = 0.009). No significant differences in the change of PTSD scores were found between the groups (*F*(2) = 2.22; *p* = 0.114). The change of PTSD and disturbances in self-organization scores and effect sizes are presented in Figure 2 and Table 2. The between-group effect size from pre-test to follow-up indicated a large intervention effect on the reduction of disturbances in self-organization score. Additionally, the within-group effect size from pre-test to post-test and from pre-test to follow-up indicated a large decrease in PTSD in the intervention group.

**TABLE 2:** Within-group effect sizes.

	Group	Pre-test – post-test *d* [95% CI]	Post-test – follow-up *d* [95% CI]	Pre-test – follow-up *d* [95% CI]
PTSD	IG	−0.90 [−1.60; −0.19]	0.00 [−0.67; 0.67]	−0.89 [−1.60; −0.19]
	WL	−0.27 [−0.73; 0.20]	−0.22 [−0.68; 0.25]	−0.45 [−0.92; 0.01]
DSO	IG	−0.54 [−1.22; 0.14]	−0.03 [−0.70; 0.64]	−0.55 [−1.24; 0.13]
	WL	0.03 [−0.43; 0.49]	0.18 [−0.28; 0.64]	0.21 [−0.25; 0.68]
Re	IG	−0.44 [−1.12; 0.24]	−0.10 [−0.78; 0.57]	−0.55 [−1.23; 0.14]
	WL	−0.14 [−0.61; 0.32]	−0.33 [−0.80; 0.13]	−0.47 [−0.94; 0.00]
Av	IG	−0.87 [−1.57; −0.16]	0.21 [−0.46; 0.89]	−0.72 [−1.41; −0.03]
	WL	−0.20 [−0.66; 0.27]	−0.01 [−0.47; 0.45]	−0.20 [−0.67; 0.26]
SoT	IG	−0.85 [−1.56; −0.15]	−0.10 [−0.77; 0.57]	−0.96 [−1.67; −0.25]
	WL	−0.29 [−0.75; 0.17]	−0.16 [−0.63; 0.30]	−0.41 [−0.88; 0.05]
AD	IG	−0.37 [−1.05; 0.31]	0.25 [−0.42; 0.93]	−0.12 [−0.80; 0.55]
	WL	−0.15 [−0.62; 0.31]	0.42 [−0.05; 0.88]	0.24 [−0.23; 0.70]
NSC	IG	−0.51 [−1.20; 0.17]	−0.11 [−0.79; 0.56]	−0.60 [−1.29; 0.09]
	WL	0.05 [−0.41; 0.51]	−0.05 [−0.51; 0.42]	0.01 [−0.45; 0.47]
DR	IG	−0.42 [−1.10; 0.26]	−0.17 [−0.84; 0.51]	−0.60 [−1.28; 0.09]
	WL	0.12 [−0.34; 0.59]	0.10 [−0.36; 0.57]	0.23 [−0.23; 0.69]

*IG, intervention group; WL, waiting list control group; PTSD, posttraumatic stress disorder; DSO, disturbances in self-organization; Re, reexperiencing; Av, avoidance; SoT, sense of threat; AD, affect dysregulation; NSC, negative self-concept; DR, disturbances in relationships.*

Separate repeated measures MANOVA analyses of the PTSD symptom clusters (Re-experiencing, Avoidance, and Sense of Threat) and disturbances in self-organization symptom clusters (Affective Dysregulation, Negative Self-Concept, and Disturbances in Relationships) were performed. At the multivariate level, the analyses revealed no significant difference in change of the PTSD symptom clusters over time between the intervention group and waiting list control group (*F*(6, 200) = 0.96; *p* = 0.451; Wilks‘ λ = 0.95). Likewise, no significant differences in change of the PTSD symptom clusters were found among the two groups at the univariate level (Re-experiencing: *F*(2) = 0.54; *p* = 0.584; Avoidance: *F*(2) = 2.04; *p* = 0.136; Sense of Threat: *F*(2) = 1.93; *p* = 0.151). The change of PTSD symptom clusters and effect sizes are presented in Figure 2 and Table 2. There were no between-group effects from pre-test to follow-up. The within-group effect sizes from pre-test to post-test indicated a large decrease in Avoidance and Sense of Threat symptoms in the intervention group. Also, a moderate decrease in Avoidance symptoms and a large decrease in Sense of Threat symptoms were observed in the intervention group, and a small decrease in Re-experiencing symptoms was observed in the waiting list control group from pre-test to follow-up.

The analyses revealed no significant difference in change of disturbances in self-organization symptom clusters over time between the intervention group and waiting list control group at the multivariate level (*F*(6, 200) = 1.90; *p* = 0.083; Wilks‘ λ = 0.90). At the univariate level, we observed a significantly higher decrease of Negative Self-Concept (*F*(2) = 4.20; *p* = 0.019) and Disturbances in Relationships (*F*(2) = 3.39; *p* = 0.038) in the intervention group compared to the waiting list control group. No significant differences in change of Affective Dysregulation (*F*(2) = 1.03; *p* = 0.362) were observed among the two groups. The change of disturbances in self-organization symptom clusters and effect sizes are presented in Figure 2 and Table 2. Between-group effect sizes from pre-test to follow-up indicated moderate intervention effect on the reduction of Negative Self-Concept symptoms and large intervention effect on the reduction of Disturbances in Relationships symptoms. No within-group effects were observed.

## Discussion

The current study adds to the growing body of literature showing that mindfulness-based internet interventions can benefit individuals exposed to traumatic events. The findings of the study expand data from the previous research, which showed that online mindfulness-based intervention was effective for CPTSD disturbances in self-organization symptoms (16), revealing that most intervention effects sustain 3 months after the end of the intervention. Disturbances in self-organization symptoms, specifically, Negative Self-Concept and Disturbances in Relationships, remained decreased in 3 months as intervention group and waiting list control group comparison revealed.

To the best of our knowledge, no previous RCTs have explored follow-up effects of internet-delivered interventions based on mindfulness principles for individuals experiencing high levels of traumatic stress. The findings of the current study are in line with other studies on mindfulness-based face-to-face interventions. Previous studies revealed a potential of mindfulness-based interventions as having lasting effects on posttraumatic stress (15, 20–23). Based on our findings, it seems that internet-delivered mindfulness-based intervention is a promising option with potentially sustaining effects. This could be relevant specifically for trauma-exposed individuals who cannot access professional psychological treatment due to existing barriers to face-to-face therapy (5). Also, it is optimistic news in the face of the COVID-19 pandemic, which has dramatically changed the understanding of providing psychological interventions by introducing the shift toward internet-delivered psychological services.

The key finding of the current study was that in 3 months, disturbances in self-organization symptoms, which are prerequisite to the new ICD-11 diagnosis of CPTSD, remained decreased. As the CPTSD is a new diagnosis raising a lot of debates about what the best treatment approach for this condition is (8), our findings are in line with the idea that for CPTSD, multicomponent therapies might be an approach that could improve the outcomes (9). Our study showed that online mindfulness-based therapy could help trauma-exposed persons to see themselves more positively and connect with others in an emotionally closer manner. Previously, it has been suggested that mindfulness-based therapies could decrease psychological arousal, increase attentional control, and foster acceptance of unwanted experiences (19). It is possible that it could be applied not only to PTSD symptoms but also could be beneficial for disturbances in self-organization symptoms in a way that practicing mindfulness could increase self-regulation capacity, which could positively impact self-concept and quality of interpersonal relationships. We assume that the same mechanisms of decreasing psychological arousal, increasing attentional control, and fostering acceptance of adverse feelings, thoughts, memories, experiences etc., could play an important role in disturbances in self-organization symptoms. And it seems that these changes tend to remain over time. However, further studies are necessary to reveal the mechanisms underlying the identified changes. Contrary to other studies in this field, our study did not show significant sustaining effects of the intervention on PTSD symptoms. We assume that the most suitable interventions for PTSD are trauma-focused therapies that address traumatic experiences and memories explicitly through trauma exposure (33, 34) whereas mindfulness-based interventions focus on purposefully paying attention to the present moment and traumatic experiences without direct addressing can remain avoided.

The current study has several limitations that should be addressed. First, regarding the measurement of outcomes, participants’ trauma exposure, as well as PTSD and disturbances in self-organization symptoms, were assessed *via* self-report, which can lead to an overestimation or underestimation of PTSD and CPTSD symptomatology. In future studies, clinical interviews administered by a trained professional should be implemented to facilitate more accurate identification of PTSD and CPTSD symptomatology and its change over time. Second, we should have in mind the fact that not all participants retained in the study at the 3-month follow-up. Despite the similarities between the retained and dropped out participants, it is possible that the participants with better outcomes remained in the study. Also, both complete cases and intention to treat analyses could be considered in the future studies to represent this field even more accurately. Third, although the results of 3-month follow-up effects of online mindfulness-based intervention on PTSD and disturbances in self-organization are promising, the intervention comprised multiple components (such as psychoeducation and different types of mindfulness exercises), and due to the study design, it is impossible to identify which of the components contributed to the symptom reduction most. Further work is required to explore mechanisms of change in the current intervention. Moreover, we cannot be sure whether the observed effects were affected by other treatments the participants were receiving. Finally, the 3-month follow-up period is still too short of drawing conclusions regarding the stability of the therapeutic gains in the long term, and future trials should address this issue. Despite these limitations, our study yielded promising results showing that mindfulness-based internet intervention can be a viable option for reducing CPTSD symptoms with the stability of the intervention effects over several months after the intervention delivery.

## Data Availability Statement

The raw data supporting the conclusions of this article will be made available by the authors upon reasonable request.

## Ethics Statement

The studies involving human participants were reviewed and approved by Psychology Research Ethics Committee, Vilnius University. The patients/participants provided their written informed consent to participate in this study.

## Author Contributions

AD: writing – first draft, data collection, data analysis, and study design. IT-K: writing – review and editing, data collection, data analysis, and study design. GA and EK: writing – review and editing, supervision.

## Conflict of Interest

The authors declare that the research was conducted in the absence of any commercial or financial relationships that could be construed as a potential conflict of interest.

## Publisher’s Note

All claims expressed in this article are solely those of the authors and do not necessarily represent those of their affiliated organizations, or those of the publisher, the editors and the reviewers. Any product that may be evaluated in this article, or claim that may be made by its manufacturer, is not guaranteed or endorsed by the publisher.

## Supplementary Material

Supplementary Figure 1 Screenshot of the intervention.
