# Supplementary figures and images for: The Effects of Online Mindfulness-Based Intervention on Posttraumatic Stress Disorder and Complex Posttraumatic Stress Disorder Symptoms: A Randomized Controlled Trial With 3-Month Follow-Up
Source: Front Psychiatry. 2022 Mar 30;13:799259. doi: 10.3389/fpsyt.2022.799259 (PMC9006994; doi:10.3389/fpsyt.2022.799259)

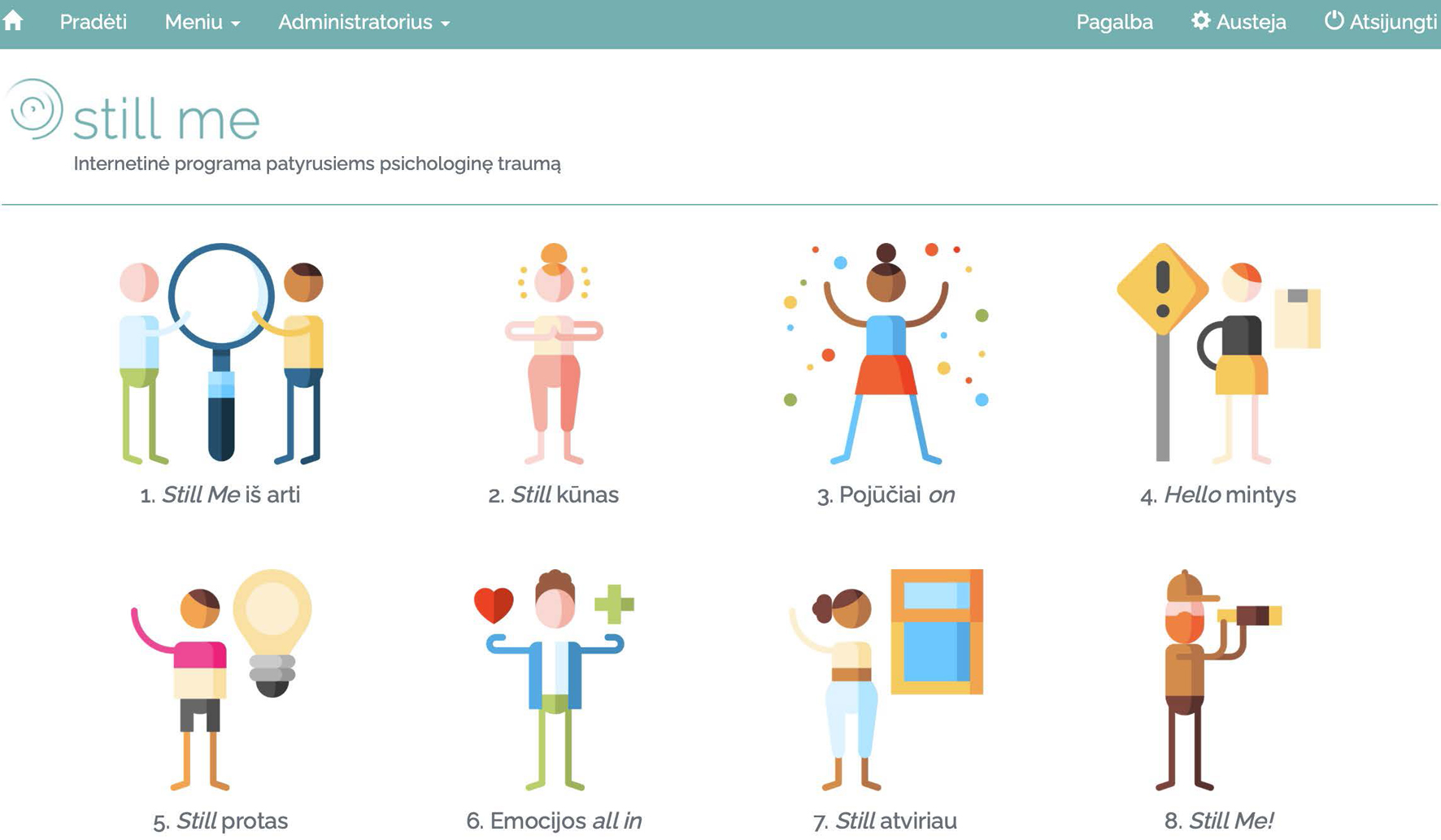

Supplement: Supplementary Figure 1 — Screenshot of the intervention. [file Image_1.jpg]
